# Tissue distribution of cloxacillin after intramammary administration in the isolated perfused bovine udder

**DOI:** 10.1186/1746-6148-6-46

**Published:** 2010-10-06

**Authors:** Manfred Kietzmann, Frank Niedorf, Jacques Gossellin

**Affiliations:** 1 Institute for Pharmacology, Toxicology, and Pharmacy, School of Veterinary Medicine, Hannover, Germany; 2 Pfizer Animal Health, Sandwich, Kent, UK

## Abstract

**Background:**

Various intramammary suspensions containing cloxacillin benzathine are registered for use in cattle as antibiotics for intramammary use at drying off. To ensure antibacterial efficacy, the glandular tissue concentration of an antimicrobial agent must be sufficient. Since the possibilities to measure concentrations in the different areas of the glandular tissue *in vivo *are very limited, it was the aim of the present study to examine the distribution of cloxacillin *in vitro *using the isolated perfused bovine udder.

**Methods:**

Mammary glands taken at slaughter from healthy lactating cows were perfused *in vitro *with warmed and gassed Tyrode solution. 600 mg cloxacillin benzathine were administered as Orbenin Extra Dry Cow by the intramammary route to six front and rear quarters each. Samples of glandular tissue - at different distances from and vertical to the teat right up to the udder base - were gathered from the treated quarters after 6 h. Perfusate was also sampled before and hourly after treatment for 6 h. The cloxacillin content of the tissue samples and perfusate samples was analysed by high performance liquid chromatography.

**Results:**

The concentration of cloxacillin in the glandular tissue of front quarters measured 6 h after administration tended to decrease with increasing vertical distance from the teat. The decrease pattern of the concentration was not quite clear in rear quarters. A considerable variation in the tissue concentrations of cloxacillin was obvious, which reflects *in vivo *conditions. The concentrations measured in the perfusate samples were below the limit of quantification at all time points, indicating limited absorption of the antibiotic from the glandular tissue.

**Conclusion:**

After intramammary administration of the dry off product containing cloxacillin benzathine concentrations of more than 0.5 μg/g (MIC) were reached in all regions of the front and rear quarters.

## Background

Various cloxacillin benzathine containing intramammary suspensions are registered for use in cattle as antibiotics for intramammary use at drying off. The drying off formulations are recommended for routine use in cows at drying off to treat existing intramammary infections and to provide prolonged protection against new infections during the dry period.

Cloxacillin is known as an effective agent against penicillin-resistant and sensitive staphylococci (*Staphylococcus aureus*), the most prevalent Gram positive pathogen involved in persistent udder infections, which are targeted by the drying off treatment. In a recent pan-European survey, an MIC_90 _of 0.5 μg/mL against *Staphylococcus aureus *(148 strains, isolated from cattle with mastitis in different European countries) was reported [15].

To ensure antibacterial efficacy, the glandular tissue concentration of an antimicrobial agent must be sufficient. For ethical and practical reasons, the local availability of active compounds *in vivo *is usually only assessed by sampling milk and blood [1,10,11]. Since the concentrations in the different areas of the glandular tissue cannot be estimated using these methods, the purpose of the study was to examine the distribution of cloxacillin was examined *in vitro *after intramammary administration using the isolated perfused bovine udder as previously described [8,4,5].

## Methods

### Test formulations

Injectors containing 600 mg cloxacillin benzathine (formulated as Orbenin Extra Dry Cow, Pfizer Animal Health, Sandwich, UK) were used. Orbenin Extra Dry Cow is approved for intramammary administration to cows at drying off.

### Isolated perfused bovine udder

As described in detail by Kietzmann et al. [8], medium sized udders with a symmetric shape and a teat length of 6-8 cm from healthy German Black Pied cows that were lactating prior to slaughter were used. Each cow's udder was examined prior to slaughter by palpation of the quarters and inspection of the milk to check for the absence of clinical mastitis. Within 10 - 15 min post-slaughter, blood clots in the vessels of the glands were cleared using 1 L heparinised Tyrode solution per udder half. Thereafter, the udders were transported for 20 min in a plastic tub to the laboratory. Within a few minutes after arrival at the laboratory, the udder was fixed in a "natural" position to a metal frame using the proximally inserting skin and suspensory ligament. Within a few minutes, the large arteries of the udder were supplied with the perfusion fluid delivered via silicone tubes. The larger veins were also cannulated to allow sampling and removal of the perfusate, whereas the smaller veins were closed using artery forceps. Six front and rear quarters from six udder udders were used.

Each udder half was perfused with 90 - 120 mL/min of Tyrode solution (136.8 mmol/L NaCl, 2.7 mmol/L KCl, 1.8 mmol/L CaCl_2 _× 2H_2_O, 1.05 mmol/L MgCl_2 _× 6H_2_O, 0.416 mmol/L NaH_2_PO_4 _× 2H_2_O, 11.9 mmol/L NaHCO_3 _and 5.5 mmol/L D(+)-glucose × 1H_2_O; 39°C) gassed with carbogen (95% O_2_, 5% CO_2_) using a peristaltic pump. The mammary glands were milked over about 5 minutes during a 30 min equilibration phase. The viability of the perfused udders was controlled using biochemical parameters such as lactate dehydrogenase(LDH)-activity, glucose consumption and lactate production in the perfusate [4,5,8]. The perfusate flux was about 90-120 mL/minute per udder half.

### Study design

After reaching a physiological like status during the equilibration period, one intramammary syringe of Orbenin Extra Dry Cow each was administered via the teat canal and massaged into the glandular cistern of one front and one rear quarter of the same side of the udder. After 6 h, four gland tissue samples were taken from the central region of the treated quarters (front quarter: 4, 8, 12, 16 cm distance to the teat, rear quarter: 5, 10, 15, 20 cm distance to the teat). Perfusate was sampled before and hourly after treatment for six h. Perfusate and tissue samples were stored frozen at -20°C.

### Analysis of cloxacillin concentrations

Concentrations of cloxacillin in glandular tissue and in perfusate samples were analysed using a high performance liquid chromatography (HPLC) method.

Tissue extraction. Samples of glandular tissue (1 g) were spiked with 10 mL of an internal standard stock solution [1.0 g/l oxacillin (Sigma, Deisenhofen, Germany) for cloxacillin quantification and filled up to a total volume of 4 mL with Soerensen phosphate buffer [33 mmol/l KH_2_PO_4_/Na_2_HPO_4 _(Merck, Darmstadt, Germany) at pH 6.8 for cloxacillin extraction. Soerensen phosphate buffer was also used in all further steps of extraction. The tissue was homogenized at 20 000 rpm at 4°C for 90 s (Ultra Turrax^®^, Janke and Kunkel, Staufen, Germany). After centrifugation at 23000 g at 4°C for 5 min, a supernatant fluid free of visible particles was obtained. For the following fluid-fluid extraction and detection of oxacillin by high pressure liquid chromatography (HPLC), a method of Schadewinkel-Scherkl [13] was modified. 1.5 mL of the prepared samples was added with 3 mL dichloromethane (Merck, Darmstadt, Germany) and immediately after acidifying with 100 μl 0.5 mol/l sulphuric acid was moderately shaken for 20 s. This was followed by centrifugation at 1500 g at 4°C for 10 min. Then 2 mL of the lower organic phase was intensively mixed with 1 mL phosphate buffer for 20 s and centrifuged again and the supernatant was separated for analysis.

HPLC analysis. Sub-samples of 100 μl were injected by an autosampler (Model 508, Beckman, Fullerton, CA, USA) and chromatographed at 40°C on a HPLC column (LiChroCART^® ^250-4 mm, LiChrospher^® ^100 RP-18e (5 μm), Merck, Darmstadt, Germany) combined with a guard column (LiChroCART^® ^4-4 mm, LiChrospher^® ^100 RP-18e (5 μm), Merck, Darmstadt, Germany). The eluent for cloxacillin analysis [phosphate buffer (33 mmol/l KH_2_PO_4_/Na_2_HPO_4_, pH 2.6)/acetonitrile (Roth, Karlsruhe, Germany) 66 : 34] was pumped at a flow rate of 1.5 mL/min (Model 126, Beckman, Fullerton, CA, USA). Cloxacillin was detected using ultraviolet light (Model 166, Beckman, Fullerton, CA, USA) at a wavelength of 210 nm. The areas under the curves were directly integrated (Software 32 Karat 5.0, Beckman, Fullerton, CA, USA).

Analysis validation. For the calculation of experimental sample concentrations, untreated udder matrix was spiked with the analyte [5 μg/g tissue]. Calibration curves were in the range of 0.5 to 5 and 5 to 400 μg cloxacillin/g tissue. The spiked samples were extracted and analysed under the same conditions as the experimental samples to create regression lines. The recovery was 91.4 ± 12.4% for cloxacillin. The limit of quantification was 0.56 μg/g. Intraday and interday precision were below 20%.

## Results

**Figure 1:**
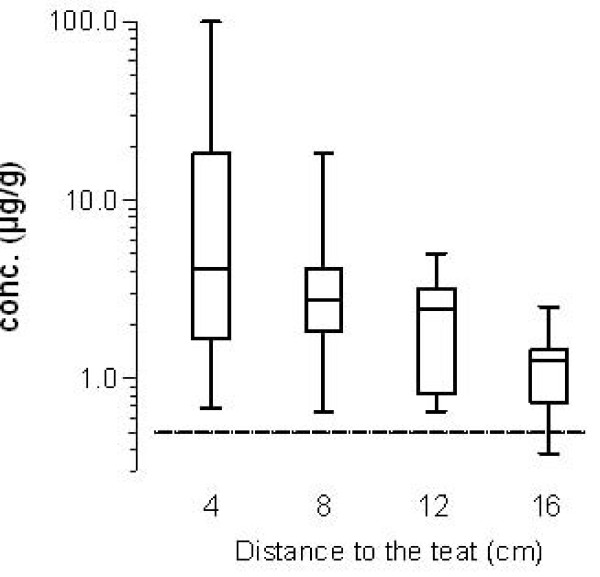
**Cloxacillin concentration (μg/g tissue) in the glandular tissue of front quarters of the isolated perfused bovine udder measured at constant vertical distances from the teat base 6 h after the intramammary administration of 600 mg cloxacillin benzathine (Orbenin™ extra dry cow) per quarter**. Data are given as box plots from six single experiments. The dotted line shows the MIC_90 _of 0.5 μg/mL against *Staphylococcus aureus*.

**Figure 2:**
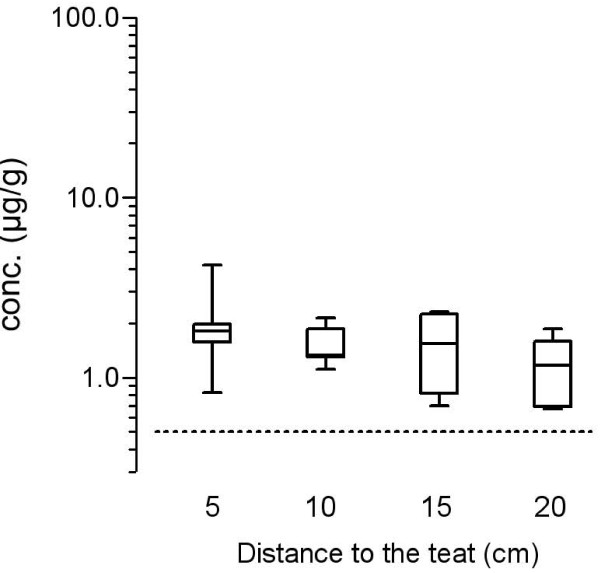
**Cloxacillin concentration (μg/g tissue) in the glandular tissue of rear quarters of the isolated perfused bovine udder measured at constant vertical distances from the teat base 6 h after the intramammary administration of 600 mg cloxacillin benzathine (Orbenin™ extra dry cow) per quarter**. Data are given as box plots from six single experiments. The dotted line shows the MIC_90 _of 0.5 μg/mL against *Staphylococcus aureus*.

The concentration of cloxacillin in the glandular tissue of all front quarters measured 6 h after administration tended to decrease with increasing vertical distance from the teat. Figure 1 shows this on a logarithmic scale. The decreasing pattern of the concentration could be explained by the high variation of the measured concentrations in the teat region. Especially in one udder quarter, a very high concentration of cloxacillin was measured near the teat. Starting with 4.1 μg/g near the teat (median), the concentration declined to 2.8, 2, 4 and 1.7 μg/g at distances of 8, 12 and 16 cm to the teat. At the base of five udder quarters, the cloxacillin concentration was between 0.73 and 2.52 μg/g while the cloxacillin was below the limit of quantification in one udder quarter. In contrast to the front quarters, the decrease of the concentration was not quite clear in rear quarters (figure 2). The cloxacillin concentration was measured between 0.67 and 4.2 μg/g in all tissue samples. Taken together, a tendency to have a quite even good distribution trough the gland tissue was obvious.

The concentrations measured in the perfusate samples were below the limit of quantification, which indicates low absorption from the glandular tissue into the systemic circulation.

## Discussion

The isolated perfused bovine udder has already been shown to be a suitable *in vitro *model for studying the pharmacokinetics of substances administered by the intramammary route and by simulation of the systemic route [4,5]. There are only few published comparative studies on the distribution of active compounds in the udder tissue *in vivo *[3]. Although it is not yet clear whether the isolated perfused bovine udder accurately reflects the situation *in vivo*, studies in the isolated perfused bovine udder offer the possibility to obtain principle knowledge about the distribution in the gland tissue as well as an answer to the question if sufficient antibacterial concentrations may be reached. It has to be considered that the study was performed using udders from slaughtered lactating cows. Because dry off formulations are used in the beginning of the dry off period, the results reflect the physiological situation because milk production is not declined at drying off.

Given the inherent variability of the measured concentration between udders which is consistent with other studies [3], the differences in cloxacillin concentrations observed in this study between various locations in the gland tissue reflect a good distribution in all regions of the mammary glands. Beyond the high variability of the data, individual results show that sufficient antibacterial concentrations above an MIC of 0.5 μg/g were reached.

The good distribution is probably related to the low particle size, afforded by the bead milling process of the formulation (Dynomill process), which ensures homogenicity of the formulation [2]. So, a concentration of more than 0.5 μg/g was reached also in the region of the udder base. In the present study, cloxacillin was measurable in all tissue samples of isolated perfused bovine udders which were treated intramammary with cloxacillin benzathine (Orbenin Extra Dry Cow) with the exception of one sample (front quarter at the base of the udder).

Following the administration of any intramammary formulation, the concentrations of drugs in the glandular tissue near the teat (cisternal area) are expected to be higher than at the base of the udder [3]. In the present study, this fact is confirmed in the front quarters but not in the rear quarters. Ehinger and Kietzmann [3] measured the cloxacillin concentration three hours after administration, while the samples were taken after six hours in the present study. Therefore, the trend to decrease seems to be lower in the present study than in the previous study where tissue samples were taken earlier. Nevertheless, the cloxacillin concentration was above 0.5 μg/g in all investigated regions of the rear quarters of the udder tissue. For *Staphylococcus aureus *and also for *Streptococcus uberis *cultured from cattle with mastitis, MIC_90 _values of 0.5 μg/mL for cloxacillin have been reported in a recent pan-European survey [12,15]. It can be concluded that potential effective concentrations can be reached in all regions of the front and rear quarters of the udder using the tested formulation containing 600 mg cloxacillin benzathine, although it has to be considered that additional factors may affect the antibacterial efficacy such as protein binding, osmotic pressure and inhibitory substances. The range of the measured tissue concentrations is comparable with results of Ehinger und Kietzmann [4,5], who studied the tissue distribution of oxacillin after intramammary administration of a formulation designated for use in lactating cows.

In the present study, there was considerable variation in the tissue concentrations of cloxacillin. This is in line with the findings of previous studies for benzylpenicillin, oxacillin, ampicillin, cefquinome and marbofloxacin [4-6,9]. This is probably due to an individual variation between healthy bovine udders, such as differences in glandular size, which also appears frequently to be correlated with the volume of secretion and enhance dilution of the active compound in the secreted milk [3]. So, this variability could reflect a normal situation in the field. The present study was conducted in a relatively small number of mammary glands (six front and rear quarters each) and therefore no statistical analysis was possible. The number of perfused udders used in the present study was not increased to allow the performance of additional statistical analysis, because the variation of the data reflects the real situation *in vivo*.

Sampling from different regions of the udder is very important when assessing the pharmacokinetics of a drug, with four locations per quarter representing the minimum, since antibiotics distribute more unevenly in glandular tissue [7] than is assumed based on milk analysis [3]. When measuring the drug concentration in milk samples from treated cows, this information can not be obtained. In contrast, the isolated perfused bovine udder allows obtaining this information without the ethical limitations of an *in vivo *study. Differences in vehicle and especially in the particle size of the suspended active principles have an influence on the distribution of antibiotics administered locally into the bovine udder [4,5,14]. Best tissue distribution rates are expected when the active compound is administered in a formulation with homogeneously distributed particles of low size, which is the case for Orbenin Extra Dry Cow, where the low particle size is obtained by the bead milling process of the formulation (Dynomill process).

This is the first study that shows antibacterial concentrations in udder tissue after intramammary administration of a dry off product containing cloxacillin measured six h after administration, concentrations of more than 0.5 μg/g were reached in all regions of the front and rear quarters. Studies in the isolated perfused bovine udder can help therefore also to screen new candidate formulations for intramammary administration to select those that will be suitable for testing *in vivo *as it was demonstrated by Ehinger and Kietzmann [5].

## Competing interests

JG is an employee of Pfizer Animal Health, the producer of the studied drug formulation. The authors declare that they have no competing interests.

## Authors' contributions

MK participated in the design of the study, organised the experimental work and wrote the draft of the manuscript. FN validated the analytical method and performed the analytical measurement. JG was involved in the design of the study and participated in the statistical analysis. All authors read and approved the final manuscript.
